# Gut Microbiome in Children with Congenital Heart Disease After Cardiopulmonary Bypass Surgery (GuMiBear Study)

**DOI:** 10.1007/s00246-024-03634-2

**Published:** 2024-08-22

**Authors:** Fatma Koc, Claire Magner, Kiera Murphy, Sean T. Kelleher, Mong H. Tan, Molly O’Toole, Dominic Jenkins, Jordan Boyle, Marie Lavelle, Niamh Maguire, Paul R. Ross, Catherine Stanton, Colin J. McMahon

**Affiliations:** 1 APC Microbiome Ireland, University College Cork, Cork, Ireland; 2 Teagasc Food Research Centre, Moorepark, Ireland; 3 School of Microbiology, University College Cork, Cork, Ireland; 4 School of Nursing, Midwifery and Health Systems, University College Dublin, Dublin, Ireland; 5 Department Paediatric Cardiology, Children’s Health Ireland at Crumlin, Dublin 12, Ireland; 6 Paediatric Intensive Care Unit, Children’s Health Ireland at Crumlin, Dublin, Ireland; 7 Laboratory, Children’s Health Ireland at Crumlin, Crumlin, Ireland; 8 School of Medicine, University College Dublin, Dublin, Ireland; 9 School of Health Professions Education (SHE), Maastricht University, Maastricht, Netherlands

**Keywords:** Congenital heart disease (CHD), Cardiopulmonary bypass (CPB), Gut microbiota, Early life

## Abstract

The gut microbiome of infants with congenital heart disease (CHD) undergoing cardiopulmonary bypass surgery (CPB) is at risk of profound alteration. The aim of this study was to examine the gut microbiome pre- and post-bypass surgery to explore potential implications of altered gut biodiversity. A prospective cohort study involving infants with CHD who underwent CPB was performed. Faecal samples were collected from infants alongside the collection of demographic and clinical data in order to examine gut microbiome changes before and after surgery. 16S rRNA sequencing analysis was performed on DNA isolated from stool samples to determine changes in gut microbiome composition. Thirty-three patients were recruited, with samples from thirteen of these available for final analysis. Compared with healthy, matched controls, at a genus level, pre-operative samples for infants with CHD demonstrated a higher relative abundance of *Escherichia-Shigella* (31% vs 2–6%) and a lower relative abundance of *Bifidobacterium* (13% vs 40–60%). In post-operative samples, the relative abundance of *Escherichia-Shigella* (35%), *Enterococcus* (11%), *Akkermansia* (6%), and *Staphylococcus* (5%) were higher than pre-op samples. One infant developed post-operative necrotising-enterocolitis (NEC). They displayed a marked abundance of the *Enterococcus* (93%) genus pre-operatively. This study demonstrates that infants with CHD have an altered gut microbiome when compared with healthy controls and there might be a possible link between an abundance of virulent species and NEC.

## Introduction

Congenital heart disease (CHD) refers to a group of structural or functional abnormalities in the heart that are present at birth [1]. CHD is the most common group of birth defects [2], having an incidence of approximately 9 per 1000 live births [3, 4]. Lesions that are dependent on blood supply through the ductus-arteriosus are a group of critical congenital heart defects that typically require intervention in the neonatal period and are fatal if untreated. They carry an incidence of 0.6/1000 live births [5]. Meanwhile, common conditions such as ventricular septal defects and tetralogy of Fallot require corrective surgery in infancy [6].

The gut microbiota, a dynamic and complex community of microorganisms residing in the gastrointestinal tract, plays a pivotal role in various aspects of human health and physiology including immune regulation and metabolism [7]. It undergoes its initial development from birth and dynamic changes occur during the first two years of life [8]. This developmental period is characterized by a rapid evolution in the composition and diversity of the gut microbiota, influenced by factors such as mode of birth, diet, genetics, and environmental exposures [9]. There are several factors that might alter the diversity of the gut microbiome in infants with CHD such as prolonged hospitalisation, mode of delivery, mode of feeding, antibiotic administration, and cardiopulmonary bypass (CPB) surgery [10, 11].

CPB is required in most surgeries for congenital heart defects performed in infancy (the notable exception being coarctation of the aorta) [12]. CPB is recognized to trigger systemic inflammation [13]. There is evidence indicating that the cytokines released after CPB resemble those released in the context of other systemic inflammatory conditions, such as sepsis and trauma [13, 14]. The inflammatory response that follows CPB with cardiac surgery is postulated to contribute to post-operative morbidity. Alterations in the gut microbiome and gut barrier of infants with CHD who have undergone CPB have been demonstrated when compared with controls in a single small study [15]. Exploring the factors contributing to the inflammatory process through the regulation of the gut microbiome, intestinal epithelial barrier dysfunction (EBD), and the resulting metabolites could enhance understanding of systemic inflammation [15, 16] which has numerous potential benefits. The inflammatory process following CPB may contribute to the development of low cardiac output syndrome (LCOS) [17] and longer aortic cross-clamp duration has been identified as a risk factor for its development [18]. Approximately 20–25% of paediatric patients, and as much as 50% of neonates, are known to experience some degree of LCOS [17, 19–22]. Among this group, those who develop LCOS tend to face higher mortality rates, prolonged stays in the intensive care unit (ICU), and extended periods of mechanical ventilation [23]. To date, treatment efforts include the widespread prophylactic use of Milrinone in the post-operative period, and in some centres the addition of post-operative corticosteroids [24, 25].

NEC is a devastating intestinal condition of infancy [10]. This condition arises when an immature or compromised gastrointestinal tract fails to maintain the mucosal barrier, facilitating the breach by bacteria, resulting in an inflammatory cascade which may lead to ischemia and perforation [26]. A robust relationship exists between gut microbiota and NEC development [27]. Studies have shown that preterm infants who develop NEC often exhibit microbial dysbalance, characterized by an imbalance in the composition of the gut microbiota [28, 29]. Studies exploring the gut microbiota of infants with CHD compared to control subjects have been limited to date [11, 15], and to our knowledge only one study has demonstrated a link between alterations in the gut microbiome and adverse clinical outcomes [11]. The relationship between NEC and the gut microbiome of infants with CHD has to date been underexplored.

In the Gut Microbiome in Children with Congenital Heart Disease after Cardiopulmonary Bypass (GuMiBear) study, the primary objective was to explore perturbations within the gut microbiota composition pre and post cardiopulmonary bypass (CPB) surgery in neonates affected by CHD when compared with healthy aged matched controls. This study aims to add to the small but growing body of literature in this domain. Full details of study design has been previously published in Magner et al. (2023) [2].

## Materials and Methods

### Study Design

**Fig. 1:**
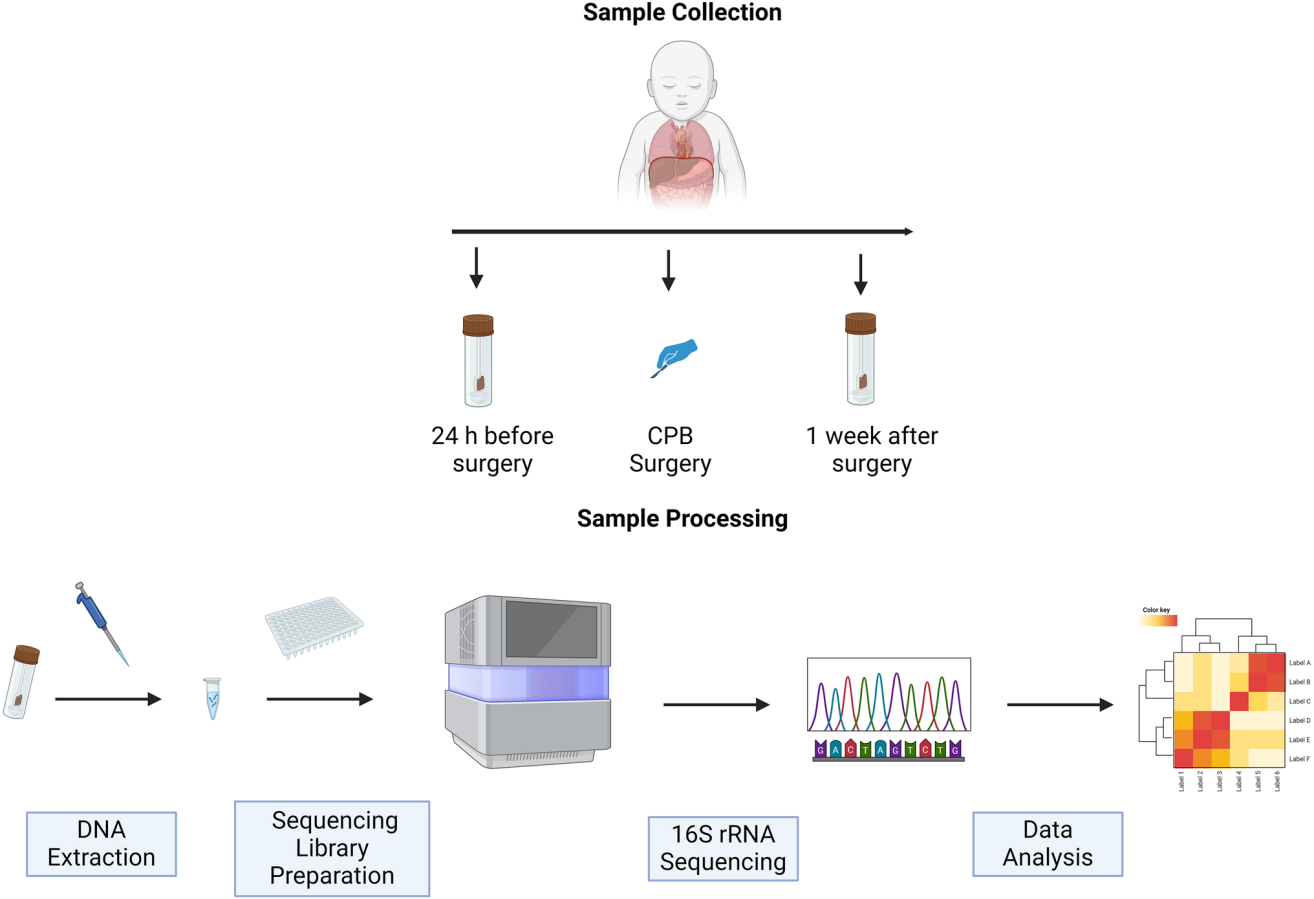
Figure illustrates the study design and sample collection process. Fecal samples were collected 24 h before surgery and one week after surgery. These samples were subjected to DNA extraction followed by 16S rRNA sequencing

This is a prospective cohort study involving infants diagnosed with CHD who underwent CPB at the National Centre for Paediatric Cardiac Surgery at Children’s Health Ireland (CHI) at Crumlin, Dublin, Ireland. In order to examine gut microbiota changes before and after surgery, faecal samples were collected from infants alongside the collection of demographic and surgical information, which includes surgical procedure performed, bypass and cross-clamp time. The collection of both were described in our previously published protocol [2]. In order to compare healthy gut microbiota with the current cohort, we used age-matched faecal samples from a previously collected cohort INFANTMET [8]. This research study is ethically approved (REC REF No: GEN/826/20). Study design and sample collection have been illustrated in Fig. 1.

### Participant Selection and Recruitment

Participants for this study were selected according to the inclusion and exclusion criteria as shown in Table 1 [2].

**Table 1:** Inclusion and exclusion criteria

Inclusion criteria	Exclusion criteria
	Stillbirth or live birth where the baby is born alive but dies shortly after
Infants diagnosed with CHD and scheduled for surgery involving CPB	Infants who are born healthy with no underlying illness, syndrome, or chronic disease
Infants born in Ireland to allow sample follow-up	Participation in another study
Ability of the participant’s parent/carers (in the investigator’s opinion) to comprehend the full nature and purpose of the study	Infants not undergoing surgery involving CPB
Consent to participate in the study and willingness to comply with the protocol and study restrictions by the participant’s parent/carers	Infants where parents/ carers do not give consent to participate in the study
	Gastrointestinal pathology or intestinal surgery, excluding gastrostomy tube

*CHD* congenital heart disease, *CPB* cardiopulmonary bypass

### Sample Collection

Faecal samples were collected from participants pre-op (24 h before surgery) and post-op (1 week after the surgery) to examine gut microbiota composition. Faecal samples were collected in the Department of Cardiology, Children's Health Ireland (CHI) at Crumlin Hospital, Ireland by either the bedside nurse or the parent/caregivers and transferred to the laboratory upon receipt and preserved at -80 °C. The samples were then transferred to Teagasc Moorepark Research Centre, Ireland on dry ice in order to process for 16S rRNA sequencing analysis. Faecal samples were stored at − 80 °C until processing for microbiota analysis.

### DNA Extraction

DNA extraction from each faecal sample was performed using a repeated bead-beading method according to Yu and Morrison, 2004 [30]. Approximately 200 mg faecal sample was weighed in a sterile 2 mL screw cap tube containing different size of beads. The extraction was performed according to the previously published protocol [31].

### 16S rRNA Sequencing Library Preparation

DNA extracts were prepared according to Illumina 16S Metagenomics Sequencing Library Preparation guidelines [31]. All amplicons were sequenced at the Teagasc Next Generation DNA Sequencing Facility using the Illumina MiSeq platform with a MiSeq Reagent Kit v3 (Illumina, Inc. San Diego, USA) according to a previously described protocol [31].

### Bioinformatics and Statistical Analysis

Sequenced reads were processed using the DADA2 pipeline, version 116 [32]. Reads were trimmed and filtered using the following parameters; maxN = 0, maxEE = c (2, 2), rm.phix = TRUE, truncQ = 2, trimLeft = c (17, 21), truncLen = c (280,210) and. maxN = 0. The core sample inference algorithm was applied to the filtered and trimmed sequence data. An amplicon sequence variant (ASV) table was created and chimeric sequences were identified and removed. Taxonomy was assigned to the sequence variants using the IDTAXA taxonomic classification method via the DECIPHER Bioconductor package and the SILVA SSU r138 2019 database (downloaded November 2022) [33, 34]. Data tables produced by the DADA2 pipeline were imported into the R package phyloseq and a phyloseq object was created for further analysis [35]. The decontam R package was used to identify and remove putative contaminating ASVs using prevalence-based method. The prevalence of each sequence feature was compared to the prevalence in negative control (DNA extraction blank) to identify contaminants using the function "isContaminant (ps, method = "prevalence", neg = "is.neg", threshold = 0.35)" [36].

Alpha diversity refers to the diversity of species within a particular habitat or ecosystem. It provides insight into the richness and evenness of species within a single sample or location [37]. Alpha diversity was examined using Shannon, Simpson, and Chao1 indices. The non-parametric Wilcoxon-Rank Sum statistical test (R function “pairwise.wilcox.test”) was used to assess the statistical significance of differences in alpha diversity indices. The Holm method was applied to adjust p values for multiple tests. Beta diversity quantifies the variation in species composition between different habitats or locations within a larger ecosystem, providing insight into the degree of similarity or dissimilarity among communities [38]. Beta diversity was measured using the Aitchison distance by applying Principal Component Analysis (PCoA) to the centred log-ratio (CLR) transformed counts. Permutational multivariate analysis of variance (PERMANOVA) was used to examine if samples cluster beyond that expected by sampling variability using the adonis () function in vegan [39]. Differentially abundant features in microbiome data were tested using MaAsLin 2 [40]. The “fixedeffects” function used surgical information (pre and post-op), Benjamini Hochberg method was applied for p value correction. The p and q values were kept at default as *p* < 0.05 and *q* < 0.25 were considered statistically significant.

Demographic information, clinical and surgical data were analysed using IBM SPSS Statistics (version 29.0.1.0). The Shapiro Wilk test was to evaluate if data set was normally distributed and Student *t* test was used to compare groups which were normally distributed. Mann Whitney *U* test was used to evaluate any significant changes pre- and post op samples.

## Results

### Enrolment

**Fig. 2:**
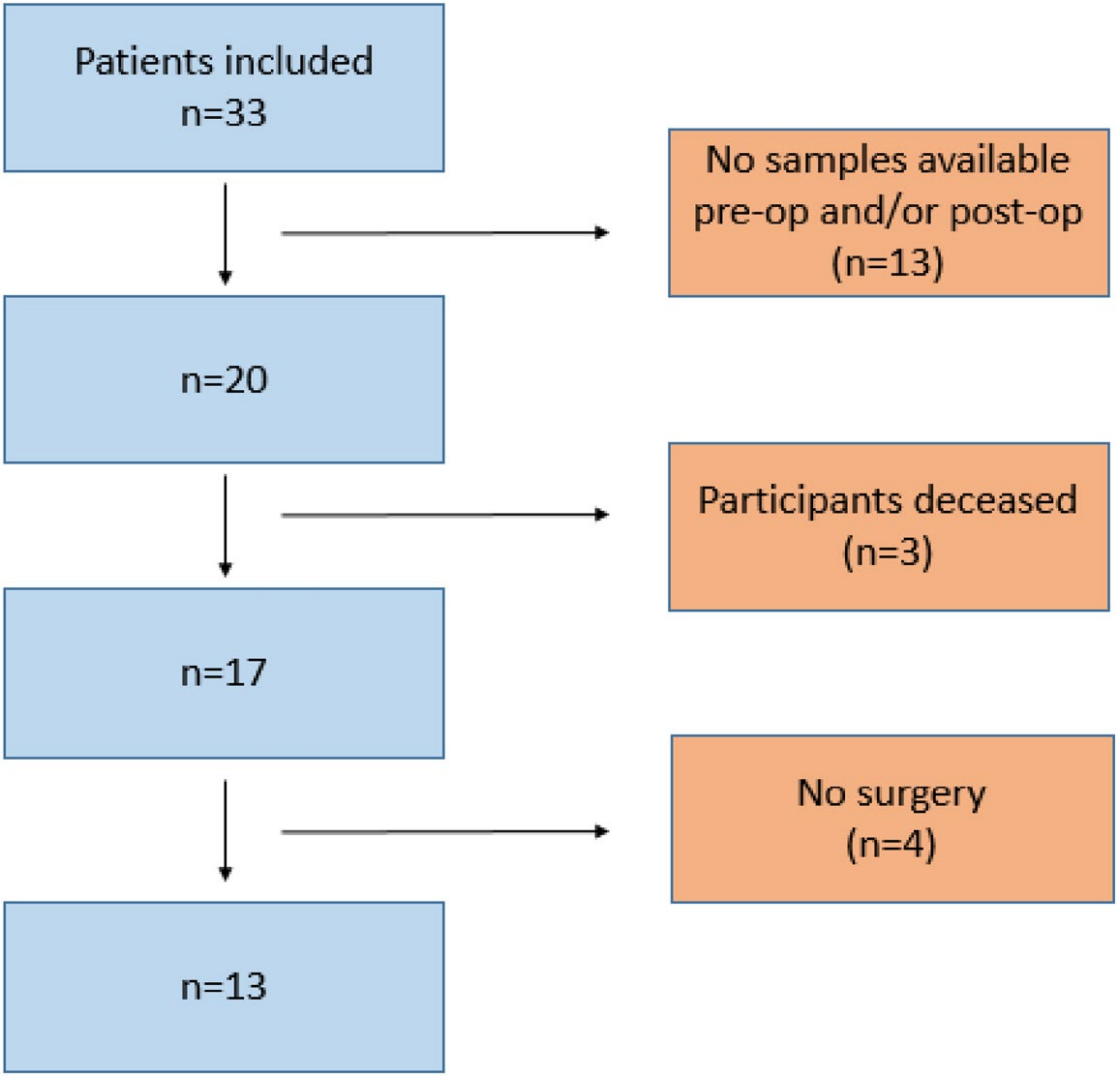
Study enrolment process. Patients reviewed according to inclusion and exclusion criteria. There was no available faecal sample for thirteen infants. Three infants were deceased during the study and four infants had no surgery

A total of thirty three patients who were diagnosed with CHD were recruited to the study (Fig. 2).

A total of thirteen eligible infants with pre-op and post op samples were finally included. Data from thirteen matched (gestational age, delivery mode, gender and sampling age) infants from the INFANTMET Study were used as healthy control comparison [8]. INFANTMET compared the gut microbiota development of breastfed infants born via C-section or vaginally at full –term or preterm at Cork University Maternity Hospital, Ireland. Informed written consent was obtained from the guardians of the participants.

### Demographic Information

Birth weight ranged from 1.22 kg to 4.14 kg with a mean birth weight of 2.92 ± 0.24. Two infants were pre-term with birth weight less than 2.5 kg (1.68 and 1.22 kg, respectively). Participants’ age ranged from 7 to 199 days, mean age of 86.23 ± 17.72 days. Gender comprised 8 female and 5 male infants. Gestational age ranged from 29 to 41 weeks and a median of 38 weeks. Bypass time differed for each patient ranging from 52 to 252 min with a mean of 126.58 ± 19.33 min. Cross-clamp time was between 20 and 174 min with mean of 80.76 ± 14.5 min.

The average Milrinone administration at day 1 was 0.52 ± 0.057 mcg/kg/min, day 2 was 0.45 ± 0.052 mcg/kg/min, at day 3 was 0.4 ± 0.059 mcg/kg/min. Morphine administration was 26.53 ± 2.22 mcg/kg/hour at day 1, 21.15 ± 2.66 mcg/kg/hour at day 2 and 14.3 ± 2.37 mcg/kg/hour at day 3. Median duration of stay in PICU was 4.5 ± 14.25 days. Two infants had a prolonged PICU admission (32 and 47 days), respectively resulting in a high standard deviation.

Nine infants were administered cefuroxime, one infant was given vancomycin + gentamycin, and one infant was given cefuroxime and piperacillin- tazobactam, one infant was given piperacillin-tazobactam, ceftazidime at post-op.

Five infants were fed by expressed breast milk (EBM), five infants were fed by formula and 3 infants were mixed fed (Table 2).

**Table 2:** Type of Feed after surgery

Type of Feed (*n* = 13)	
EBM (Expressed breast milk)	5
Formula	5
Both	3

### Hospitalization and Surgery

Arterial blood gas (ABG) pH and ABG pO_2_ levels were recorded before and after surgery. The average of ABG pH was 7.43 ± 0.18 and ABG pO_2_ was 93.6mmHg ± 66mmHg before the surgery. The average of ABG pH was 7.31 ± 0.07 and ABG pO_2_ was 125.1mmHg ± 64.4mmHg after the surgery.

### Microbiota Analysis

**Fig. 3:**
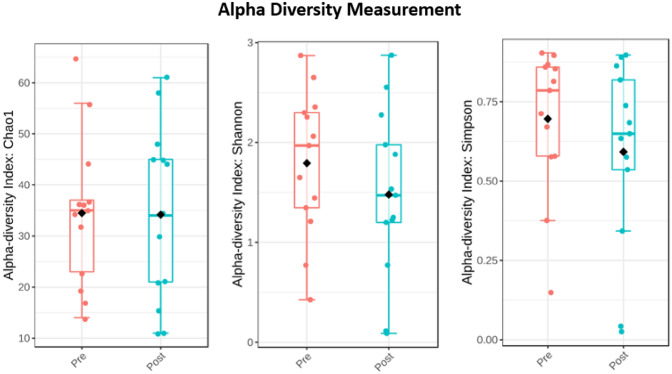
Box plots describing the Chao1, Shannon and Simpson between pre-op and post-op samples

Faecal samples were analysed pre (*n* = 13) and post operatively (*n* = 13) to evaluate gut microbiota composition. Alpha diversity was evaluated to examine richness and evenness of bacterial species within each sample (Fig. 3). No statistically significant differences in Chao1, Shannon and Simpson alpha diversity indices were found in the pre-op group compared with the post-op group.

**Fig. 4:**
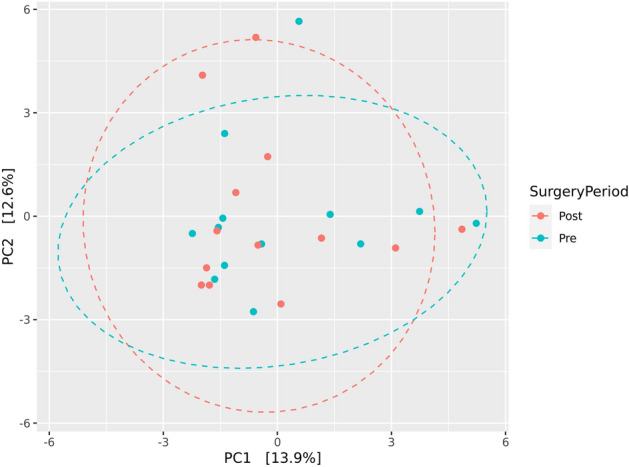
Principal component analysis (PCA) using Aitchison distance shows no significant separation between pre-op and post-op samples

Principal component analysis (PCA) was used to evaluate beta diversity in pre-op and post-op samples (Fig. 4). PERMANOVA was used to test if samples were clustering beyond the expecting variability. There was no significant separation between pre-op and post-op samples.

**Fig. 5:**
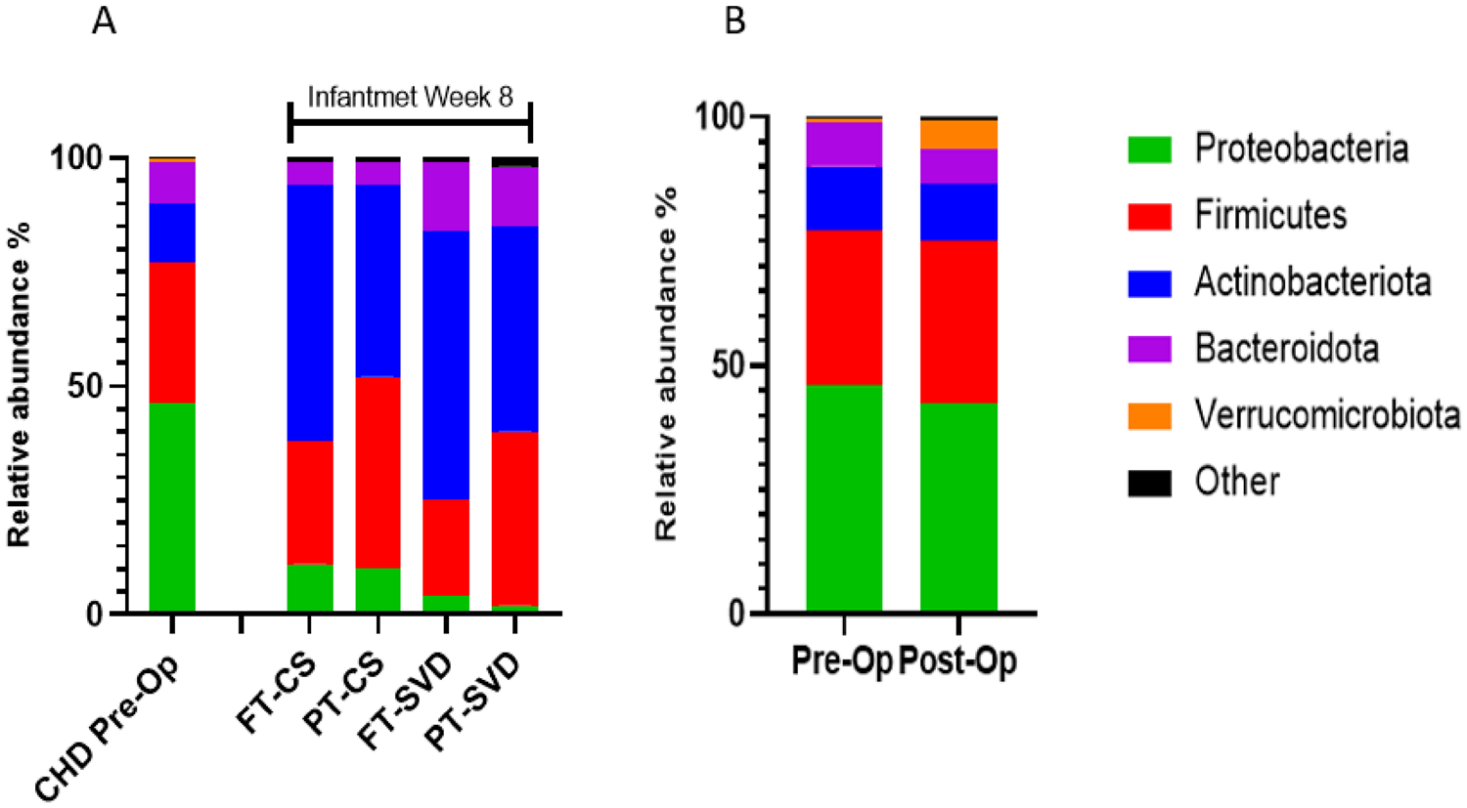
Graphic shows relative abundance at phylum level. **A** CHD Pre-Op samples from this study. FT-CS, PT-CS, FT-SVD, and PT-SVD displays samples from Infantmet cohort. **B**, the relative abundance of pre-op and post-op samples are shown

Pre-op samples were compared with INFANTMET control samples at week 8 of life [8]. Proteobacteria was found to be the most abundant phylum with 46% relative abundance followed by Firmicutes (31%), Actinobacteria (13%), and Bacteroidota (9%) in CHD pre-op samples. By contrast, in the INFANTMET samples, the most abundant phylum was Actinobacteria (Fig. 5A).

When pre-op samples were compared with post-op samples (Fig. 5B), the relative abundance of Proteobacteria, Actinobacteria, and Bacteroidota were found to be slightly reduced in post-op samples (42%, 11%, and 7%, respectively). Firmicutes and Verrucomicrobiota were slightly elevated in post-op samples (33% and 6%, respectively).

At the genus level, notable alterations in microbial composition were observed between the preoperative (pre-op) and INFANTMET samples. In the pre-op samples, *Escherichia-Shigella* (31%), *Bifidobacterium* (13%), *Veillonella* (12%), *Bacteroides* (9%), and *Enterococcus* (9%) emerged as the five most predominant genera. Furthermore, the relative abundance percentages for *Staphylococcus*, *Akkermansia*, *Lactobacillus*, *Prevotella*, *Fusobacterium*, and *Streptococcus* in the pre-op samples were 0.44%, 0.8%, 0.01%, 0.05%, 0.06%, and 2%, respectively. In contrast, the INFANTMET samples displayed a microbiota composition differing from that of CHD samples [8]. In INFANTMET samples, *Escherichia-Shigella* accounted for 2–6%, *Bifidobacterium* ranged from 40 to 60%, and *Veillonella* constituted 2% of the relative abundance.

**Fig. 6:**
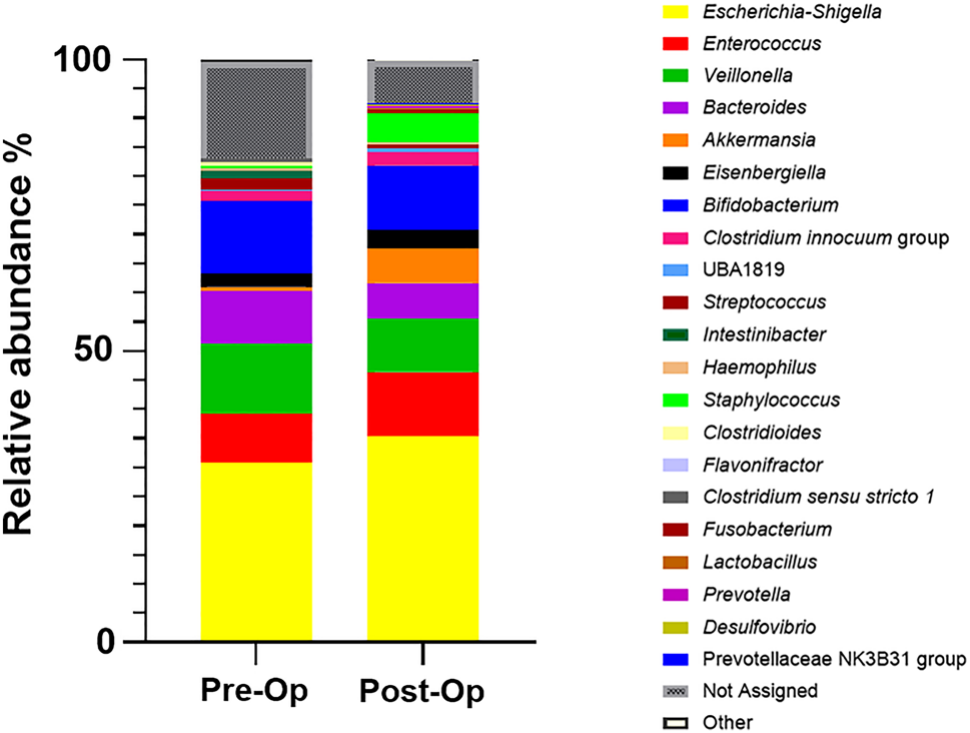
The graphic shows the relative abundance at genus level between pre-op and post-op samples

Gut microbiota composition revealed distinct differences between pre-op and post-op samples at the genus level (Fig. 6). Increased relative abundances of *Enterococcus* (11%), *Akkermansia* (6%), *Lactobacillus* (0.2%), and *Staphylococcus* (5%) were found in post-op samples. Conversely, higher relative abundances of *Escherichia-Shigella* (35%) *Veillonella* (9%), *Bacteroides* (6%), *Streptococcus* (0.5%) were observed in the post-op samples.

Furthermore, MaAsLin2 was used to determine multivariable associations between clinical metadata and microbial features. No significant differences were identified following the Benjamin-Hochberg *p* value correction (*q*-value > 0.25).

### NEC and Gut Microbiota

**Fig. 7:**
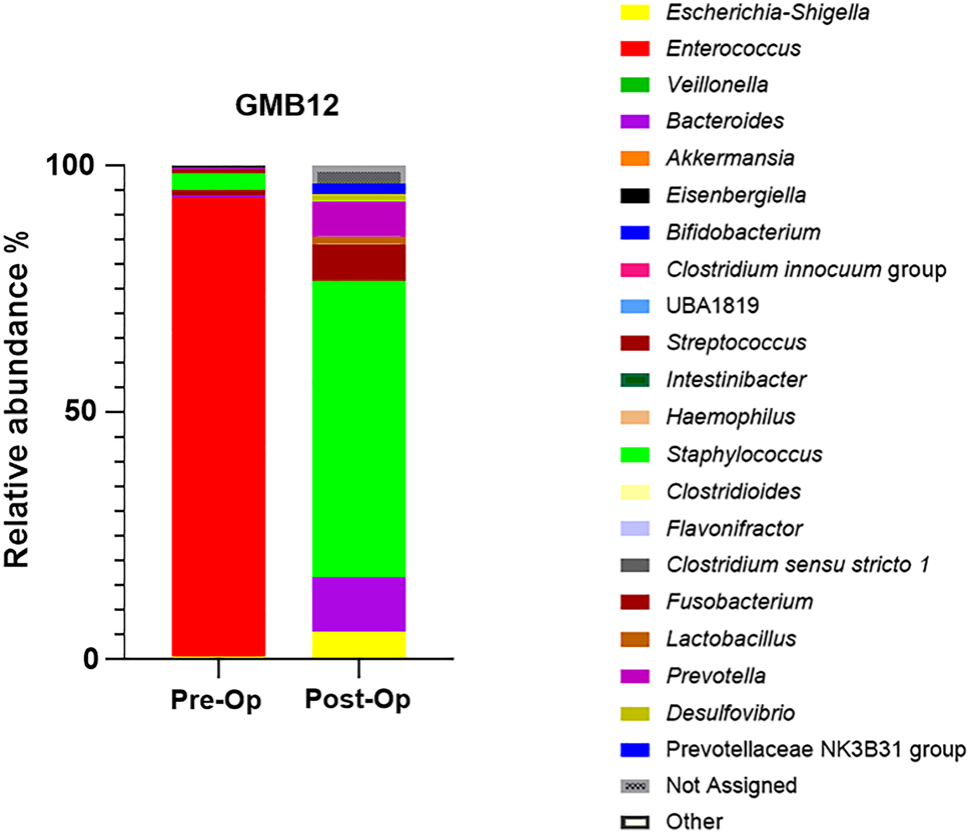
The bar chart represents relative abundance of genus levels in GMB12

One infant (GMB12) developed NEC on post-op day 3. The age of the infant was 10 days, born at 37 weeks gestation. The patient underwent an arterial switch operation, aortic arch repair, VSD closure, ASD closure and left pulmonary artery patch plasty. Infant GMB12 was administered benzylpenicillin and gentamicin before the surgery, and post-surgery, a combination of cefuroxime, gentamicin, amoxicillin, and metronidazole was given as antibiotics. Bypass duration was 249 min and cross clamp duration was 129 min. He was on ventilation for 28 days. Mode of feeding was nasogastric tube and he was fed by EBM. He was in PICU for 32 days. The relative abundance of *Enterococcus* was the most dominant genus in the pre-op sample of the infant with the relative abundance of 93% (Fig. 7). Following the surgery, *Staphylococcus* was the most abundant genus with relative abundance of 60% followed by *Bacteroides* with 10%, *Fusobacterium* with 7.4%, and *Prevotella* with 7% and *Escherichia-Shigella* with 6%. Following surgery the relative abundance of *Enterococcus* level was less than 1% (Fig. 7).

## Discussion

This prospective cohort study was conducted to explore gut microbiome alterations during CPB on infants who were diagnosed with CHD. 16S rRNA sequencing was performed on faecal samples from pre-surgery and post-surgery to explore alterations in gut microbiome. DNA samples from a previous longitudinal cohort study (INFANTMET) were used as age-matched healthy controls. One infant from the current study developed NEC three days after the surgery. The microbiome data of the infant was explained exclusively.

Alpha diversity and beta diversity did not show any significant differences between pre-op and post-op samples. However, taxonomic analysis at phylum and genus levels displayed differences between the pre-op and post-op samples based on percentages, however these differences were not statistically significant.

Before surgery, Proteobacteria was found to be the most dominant phylum in the gut microbiota of infants in this study. In INFANTMET cohort samples, the relative abundance of Proteobacteria was lower than pre-op samples in this study [8]. The increase of Proteobacteria may be driven by the abundance of *Escherichia-Shigella* genera in samples. A 2021 study, where they compared the microbiota of CHD patients with control subjects, found higher relative abundance of Proteobacteria in pre-op samples than healthy infants [15].

At the genus level, *Enterococcus, Escherichia-Shigella* and *Staphylococcus* were found to be higher in post-op samples than pre-op samples whereas *Bifidobacterium*, *Bacteroides and Veillonella* were found to be lower in post-op samples than pre-op samples. A similar trend was observed in a cohort study with lower relative abundance of *Bacteroides* in post-op than pre-op samples in infants [15].

*Escherichia* and *Shigella* are known as opportunistic pathogenic bacteria and are associated with a range of infections mainly in gastro-intestinal diseases while *Escherichia coli* can be considered as a commensal microorganism [31]. Increased levels of *Escherichia* were observed in gut dysbiosis such as small intestine bacterial overgrowth and inflammatory bowel disease [41]. *E. coli* that produces lipopolysaccharide (LPS) has the potential to induce systemic inflammation, serving as a contributing factor to epithelial barrier dysfunction implicated in various diseases including NEC [42, 43]. LPS has been shown to have an important role in NEC development [44, 45].

The relative abundance of *Akkermansia* was found to be higher in post-op samples than pre-op samples. Recent studies showed that *Akkermansia* contains a strain named *A. municiphila* which is considered as a next generation probiotic [46, 47]. Studies showed that *Akkermansia* might be a potential beneficial bacteria to reduce the risk of developing cardiovascular diseases [46].

The infant who developed NEC following the surgery had a relative abundance of 93% *Enterococcus* in their gut microbiome. There are several bacterial strains found to be related to NEC yet there is no single strain found that is solely responsible [48]. The relationship between *Enterococcus faecalis* and NEC has been previously described in the preterm population [49]. Earlier studies showed that in this population, patients who developed NEC tended to have lower percentages of *E. faecalis* than healthy controls but this did not reach statistical significance [50, 51]. More recently, specific strains of *Enterococcus faecalis* have been shown to significantly increase NEC pathology [49]. *Enterococcus* is the third most common nosocomial pathogen and rates of antibiotic resistance are increasing [52]. A recent study of neonates with CHD, demonstrated an abundance of enterococcal species, the presence of which was linked to adverse surgical outcomes and systemic inflammation [53]. While this study is not powered to fully explore outcome data, it is notable that this patient had an abundance of enterococcal species and a challenging post-operative course in addition to the development of NEC.

After surgery, *Staphylococcus* was the most abundant genus in the gut microbiome of NEC patient, with a relative abundance of 60% followed by *Bacteroides* (11%), *Fusobacterium* (7.4%) and *Prevotella* (7%). Coagulate negative *Staphylococcus* was commonly found in NEC patients’ gut microbiome and was shown to be a prominent pathogen in neonatal intensive care units and the majority cases of neonatal sepsis [54–56]. *Staphylococcus epidermidis* is likely to be a major contributor to NEC in infants [57]. *S. epidermidis* was characterized by carriage of pathogenic factors such as *icaA*, IS256, SCC*mec*, and toxins which were found to induce mucosal necrosis and haemorrhage in the bowel [58, 59]. *Staphylococcus aureus* is another species of the *Staphylococcus* genus which can damage the cell membrane via producing phenol-soluble modulins [60].

## Study Limitations

The initial composition of the gut microbiota among infants undergoing cardiac surgery in this cohort varied considerable possibly reflecting underlying differences in patient characteristics. Rapid changes in the gut microbiome occur over the first two years of life and the wide age range of our patients at time of initial sampling is one such explanation. This heterogeneity posed a challenge in examining changes in the gut microbiota pre and post operatively. Non-significant trends in the gut microbiota of infants undergoing CPB pre- and post-operatively were identified but these did not reach statistical significance. With regards to post-operative samples, there is a wide variation in antibiotic administration which varies considerably between patients rendering analysis on the impact of antibiotic administration on the gut microbiome difficult. A larger cohort may uncover statistically significant changes and may also shed light on additional influences on the gut microbiota that may contribute to the development of NEC. Furthermore, healthy controls were matched based on gestational age, delivery mode, gender, and sampling age but not feeding type. Healthy controls were exclusively breastfed, while infants with CHD may have received either breast milk or formula. This is a potential confounding factor, and the possibility that it may have contributed to the observed gut microbiome alterations cannot be excluded. Longitudinal studies are imperative to monitor gut microbiota changes over time and identify when the gut microbiota aligns with that of a healthy cohort. In addition to the gut microbiota, investigating immunological parameters in patients may offer connections between immune-microbiota interactions and the onset of NEC.

## Conclusion

This research uncovered notable differences in the gut microbiota of infants with CHD compared to healthy controls. However, in this small cohort no statistically significant differences were identified in pre- and post-operative samples. Administering probiotic strains after surgery could be a beneficial strategy to enhance infant gut health, potentially leading to improved overall well-being, reduced hospitalization duration, and a lower risk of developing NEC.

## Data Availability

No datasets were generated or analysed during the current study.
